# “I am called girl, but that doesn’t matter” -perspectives of male nurses regarding gender-related advantages and disadvantages in professional development

**DOI:** 10.1186/s12912-021-00539-w

**Published:** 2021-01-20

**Authors:** Aimei Mao, Pak Leng Cheong, Iat Kio Van, Hon Lon Tam

**Affiliations:** Kiang Wu Nursing College of Macau, Est. Repouso No.35, Macau, China

**Keywords:** Male nurses, Masculinity, Professional development, Qualitative research, Macau

## Abstract

**Background:**

Exploration of professional development experiences of male nurses can help develop evidence-based strategies to attract males into nursing. The study aims to investigate the advantages and disadvantages of being a male in nursing profession that male nurses and male nursing students experience in their professional development.

**Methods:**

A descriptive qualitative research was designed. Purposive sampling was applied and 24 males (12 nursing students and 12 clinical nurses) participated. Semi-structured individual interviews were used in data collection. Thematic analysis was used in data analysis.

**Results:**

Professional development of male nurses was related to three interplayed identities: a man, a nurse, and a minority. Three themes emerged relating to the professional development of the males: “feeling role strains” “taking perceived advantage of masculine traits” and “taking an egalitarian viewpoint”. There was no clear line between the gender-related advantages and disadvantages as factors influencing professional development can be turned by the males from barriers to facilitators.

**Conclusions:**

Male nurses perceive nursing as equally suitable for males and females and make use of masculine traits to thrive in their professional development.

## Background

The nursing workforce shortage challenges the healthcare systems in many countries. The World Health Organization (WHO) projects 32.3 million nurses/midwives in 2030 based on the current trend, while the needs-based number can be 40 million [1]. The WHO names Year 2020 “the Year of the Nurse and Midwife” to honor nursing professionals’ contributions to the society [2]. It also calls for strategies to address the risks associated with nursing workforce shortage [2]. Attention is being paid to the recruitment of males into nursing, an issue overlooked in the past.

Nursing has been portrayed as a feminine occupation ever since the appearance of Nightingale nursing training style in the mid-nineteenth century which favoured women over men to become nurses [3]. Equal education between females and males and efforts to promote equality by the wide society beyond health care arena in the past half century have seen women enter men-dominated occupations and vice versa [4, 5]. Despite the narrowed gender inequality in other occupations, such as medicine, law and business, nursing remains a women-dominated profession. In the United States, the number of male nurses in 2019 accounted for 12% of the nursing workforce; while the figure was 2.7% in 1970 [6]. In Canada, the proportion of male nurses among nurse population slowly increased from 5.7% in 2007 to 7.8% in 2016 [7].

The social norms in East Asia are much influenced by Confucianism. Caring work is traditionally considered in the eastern world to be women’s domain and men are regarded as the breadwinners of the family, doing decently paid work outside home. This prevailing social attitudes towards gender-related caring work have marginalised the males who perform nursing care. Therefore, fewer men in the eastern world join nursing than in western countries. For instance, there is approximately 6.2% of male nurses among nursing workforce in Japan [8]; in mainland China, males only account for 2.1% of the nurse population [9]. The male nurses in China are so few that they are described as “national treasures” and are generally hired by major health institutions [10].

Nurses are portrayed as humble, gentle, subordinate, empathy, etc. and masculine traits such as proactive, dominant, are not suitable for the nursing career [11, 12]. This stereotyped conception has put males under pressure in deciding to join or stay in nursing [11–13]. On the other hand, the lack of male nurses sometimes positions female nurses in an embarrassing situation in patient care [14, 15]. Y Zang [15] examined the experiences of female nurses in providing genitalia related care for male patients. Both nurses and patients felt uncomfortable with caring experiences. Other studies have similar findings that patients prefer the services from the same-sex nurses [12, 16].

Whereas men’s reluctance to joining nursing is a common phenomenon in many countries their motivations to enter nursing is shaped by gender-based ideologies in a specific social-cultural context. It is, therefore, important to explore the experiences of those men who are already in nursing profession in different social-cultural contexts. Such kind of knowledge will have implications for the nursing development not only for the specific society where the men live, but also for the global nursing community.

Macau is a special administrative region of China, along with Hong Kong. Like other regions in the world, Macau has a shortage of workforce in nursing. It is a small city with a population of 667,400 and has 2464 registered nurses, with 3.7 nurses per thousand of population [17], while in Hong Kong, the proportion of nurses per thousand of population is 7.3 [18]. The proportions of nurse and midwife/population in many other high-income countries are much higher than that in Macau, too. For example the proportion is 14.5 in the USA, 6.2 in Singapore, and 8.2 in the UK, respectively [19]. There are 270 male registered nurses in Macau, accounting for 11% of the nursing workforce [17]. While this ratio is higher than that in mainland China or Japan, it is lower than that in the USA, as mentioned before. Little attention is paid to the professional development of male nurses in Macau. Our study aims to explore the advantages and disadvantages of being a male in nursing profession perceived by male nursing students and clinical nurses in their professional development.

## Methods

### Participant recruitment

Descriptive qualitative research was designed [20]. Purposive sampling method was used. The recruitment criteria included: 1) male nursing students in a bachelor’s degree program because holding a bachelor’s degree is the requirement for professional registration for nursing in Macau; OR 2) the clinical nurses who had graduated from a bachelor’s degree program and had at least 1 year of work experience. A heterogeneous purposive sampling method was applied, which could provide a diverse range of participants relevant to the research purpose. The researchers invited potential participants from different backgrounds, including years of study or work experiences, clinical departments, origins of growth (Macau, Hong Kong, or mainland China), etc. Two nursing schools in Macau provide nursing programs in bachelor’s degree. The participants were current students or graduates from the older and larger nursing school. Therefore, while the participants came from one nursing school, they represented male nurses from different stages of professional development and different places of growth and work settings.

With the help of some nursing educators and clinical nurses whom the researchers knew of, the researchers got a list of potential participants. The researchers then approached the potential participants, explaining the purposes of the study, time contribution expected from the participants, the risks and/or benefits of participation. Confusion about the study was clarified. Should the potential participant be interested in the study, an interview arrangement with the time and place mutually convenient to both the participants and the researchers was scheduled.

Twenty-four male participants took part in the study, with 12 students and 12 clinical nurses. Among the 12 students, five were in Year One; three were in Year Two; Two were in Year Three, and the other two were in Year Four. Two students were from Hong Kong, and the other ten were locals. None of them were from Mainland China.

The 12 clinical nurses all worked as frontline nurses, who provide direct health care for patients. They had worked for 1 to 11 years, with average employment length being 5.6 years. Five of them were working at the emergency department of hospitals, with two at ICU (Intensive Care Units), two at hemodialysis units, and the other three in other areas.

### Data collection

Semi-structured, in-depth interviews are the most commonly used technique of data collection in qualitative research studies [21] and this technique was used in our study. An interview guideline was developed based on the researchers’ literature review and their long-term experiences as clinical nurses and nursing educators. The interview guideline (Table 1) allowed the interviews to focus on the questions related to the purposes of the study and, at the same time, invited flexibility to explore the emerging points during the interviewing. Probes were used to explore interesting points or clarify vague accounts. Field notes were made immediately after interviewing, recording the contexts of interviewing and the overall impressions of the interviewers about the interviewees’ performances.

**Table 1:** The interview guideline

The open-ended questions	
1. Would you please tell me what nursing means to you?	
2. How did you join nursing? Please describe the story that you chose nursing.	
3. How did the people around you (your family, your friends, and your schoolmates) react to your decision to be a nurse?	
4. What advantages did you have as a male nurse?	
5. What disadvantages did you come across as a male nurse? How did you cope with the difficulties or challenges you came across?	
6. What did you think of the public attitudes towards nurses in Macau?	
7. What is your plan in the future as a nurse?	

All the participants chose the tutorial rooms of the nursing school because the rooms were quiet. No one else was present except the participants and the researchers. The interviews lasted 23–78 min, with the average time being 45 min. Repeated information from later participants indicated saturation of the data. This means no new information emerged from the data, signifying the point to terminate further recruitment and interviews [22].

### Data analysis

All the interviews were audio-recorded with the approval of the participants. They were then transcribed verbatim. Classic thematic analysis was used which was inductive, following the four steps below [23]:

1. reading the interview transcripts several times, sometimes against the audios, to gain a thorough familiarity with the data;
2. analyzing the data line by line while assigning sections of text into meaningful units, labeling with codes;
3. identifying the relationships between codes, sorting related codes into subthemes and subthemes into themes;
4. formulating themes as the expression of the latent of the content of the texts.

Qualitative research software Nvivo11 Plus was used to facilitate the data analysis. The four team members independently coded three interviews; they then compared and discussed the coding results. Consequently, a coding framework was established. The codes were defined in the framework so that consensus on the coding could be reached among the researchers. The first author, an experienced qualitative researcher, applied the coding framework to code the remaining 21 interviews. The framework was directive rather than being rigid so that modifications could be made when new codes and themes emerged as data analysis progressed. The analytic results were agreed by all the team members.

### Ethics consideration

This study was approved by the research committee of a nursing college in Macau (reference no: 2016JAN01). It was also granted by a foundation in Macau (reference no:1962/DSDSC/2016). Confidentiality were assured as identifications of the participants and their individual responses to research questions would not be disclosed beyond the research team. The participants were told the right to withdraw from the study anytime without adverse effects imposed on them. The participants signed a consent form before the interview began.

### Rigor

The rigor of the study was assured by the measures advocated by Lincoln and Guba for qualitative research [24]. The study was conducted by a group of researchers who were all registered nurses. One of the researchers was a male nurse who had worked as a frontline nurse for several years. While the similarity of professional background between the researchers and those under scrutiny had helped interviewing and data analysis, the research team was cautious to the possible adverse effects of the similarities. Reflexivity was applied by the researchers through which the researchers consciously and critically reflected on their beliefs, values, and biases as these might influence the research process and the analytic outcomes [25]. Member check is another way to improve study credibility and in this study member check was done by sending the analytic results back to two of the participants for their feedbacks. No revisions were suggested from the two participant reviewers. Other measures included re-reading of the data to confirm the findings, thickly describing the research process, providing quotes from the interviews to explain the findings, etc.

## Results

The male participants were clear that they were a minority among nursing professionals. While they experienced perceived advantages and disadvantages in their professional development, they tried to position themselves as equal to their female counterparts. The professional development was shaped by three interplayed identities of the participants: a man, a nurse, and a minority. The nurse’s identity was paramount because, according to the participants, behaving professionally was the key to gaining trust and respect from patients and other health professionals. Being a man and being a minority exerted as both barriers and facilitators to nursing professional development.

**Fig. 1:**
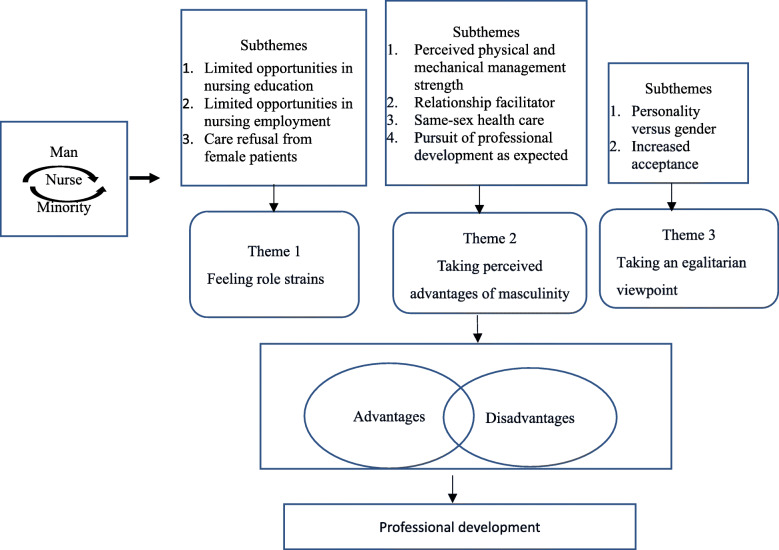
the gender-related advantages and disadvantages in professional development perceived by male nurses

Factors influencing professional development could exert their forces in competing directions, depending on the contexts of the male nurses’ professional life and their interpretation of the contexts. These factors were grouped into subthemes and the subthemes into three themes: “feeling role strains” ‘taking perceived advantage of masculinity” and “taking an egalitarian viewpoint”. Please see Fig. 1.

### Theme 1: feeling role strains

As a minority, the male nurses experienced pressure and difficulties in nursing school and health institutes. There were three subthemes relating to theme1:“limited opportunities in nursing education”, “limited opportunities in nursing employment”, and “care refusal from female patients”.

#### Limited opportunities in nursing education

Male nursing students recounted their experience of being marginalized in the group task. One participant had just finished his first year in nursing school. He felt that male students were somehow side-lined by their female team members:

*Each group usually has five or six student members and may have one or two boys. Girls have an advantage in the number. So they initiate the discussion and discuss or chat and do not see boys. We boys may have a say, but the girls decide the discussion direction.* (MN 12)

This phenomenon existed predominantly among junior students. As students advanced to seniors, they knew well who was committed to collective tasks and wanted to invite those who were willing to contribute to their groups. Male students also felt that they sometimes were difficult to find peers in laboratory practices. Again, senior male students did not see this as a problem because they had fewer basic clinical skill lab classes after the first 2 years.

#### Limited opportunities in nursing employment

Another obstacle male nurses came across was the limited clinical departments they could be employed. This was more obvious in private hospitals than in public health institutes as the former was more seriously short of the nursing workforce. The male nurses were limited to several departments: emergency department, operation theater, ICU, hemodialysis units, etc. They were usually excluded from the clinical departments where there was only one nurse on night duty, as one of the participants explained, “*It is inappropriate that one male nurse is the only nurse on the night duty*.” (MN 5).

Most of the male nurses did not mind the employment limitation. In fact, they welcomed the limitation. They generally preferred the clinical wards assigned for male nurses. A clinical nurse who graduated 1 year before and had worked in the emergent department ever since, described why he preferred the emergent department:

*After graduation, I applied for the emergency department as my first choice, the ICU as the second and the palliative care ward as the third. The first two were the departments I really wanted to go to because it is challenging to work there. I like challenging work. I think young nurses should go to these departments to gain experience.* (MN 9)

Confinement to limited clinical wards also meant stable employment; so the males could pursue specialty nursing. Those participants who were working or had worked in ICU mentioned that they chose ICU because *“There are lots of machines in the ICU, and it is a specialized nursing*”. (MN 15).

#### Care refusal from female patients

Almost all of the males had experienced being refused care services by female patients. This happened particularly when the nursing care was provided on the privacy areas of the body, for example, the perineal site, or breast area. While some of the males felt frustrated because they lost practicing opportunities, others did not see the refusals as a problem. According to the males, the refusals did not happen very often, about 4 or 5 times a year. One man who was working in a hemodialysis unit believed that there would be no embarrassments as long as the communications between male nurses and female patients went well:

*We sometimes need to insert a catheter for the patients who undergo hemodialysis. Some young women patients wear a bra. They need to take off the bra and expose their chest area. I would explain the whole procedure to them. Then I would ask whether they themselves can take off the bra. I would not take off the bra for them because they had better do it themselves. I think clear explanations are very important. They then know why they have to expose the chest. There would be no embarrassment.* (MN 11)

### Theme 2: taking perceived advantages of masculinity

Despite the restraints in the health care services mentioned above, the male participants did find some advantages as a male. There are four subthemes relating to theme 2: “perceived physical and mechanical management strength”, “relationship facilitator”, “same-sex health care”, and “pursuit of professional development as expected”.

#### Perceived physical and mechanical management strength

The apparent advantage perceived by the males was physical strength. The participants repeated that they were often asked by their female colleagues to lift the patients. One student in Year 4 just finished his one-year of clinical practice. He mentioned the advantages of male nurses in lifting patients, “*Female nurses have difficulty in lifting their patients, particularly if the patients are heavy. I would go there to give a hand*.” (MN 3). Offering helps enabled him to enjoy a good relationship with his female supervisors during the clinical studies.

Men believed that they were better at dealing with machines than women. A male nurse who worked in the hemodialysis unit articulated that there were lots of machines in the hemodialysis unit, “*I was quicker to get familiar with the machines than those females who joined the unit the same time as me. I had got the feeling that men were good at dealing with machines when I was in the operation theater. I do not know why. That is the nature of men*.” (MN 11). His viewpoints were shared by other male participants who worked in the emergency department and ICU.

#### Relationship facilitator

Despite a minority among nurse professionals, male nurses did not find it challenging to build a relationship with their female counterparts. In fact, they sensed welcome as the team members. They joked the old saying, “男女搭配, 幹活不累”(A mix of Jacks and Jills makes a tough job a breeze). One man had worked in the emergency room for 3 years, and he talked about the male role as a relationship facilitator:

*Women sometimes pay too much attention to trifles, and that can cause conflicts. If there are several men in the team, the atmosphere will be different. Men tend to be more humorous than women. If one or two female nurses burst one or two rude words to us, we just smile off.* (MN 18)

The males were sometimes asked by their female colleagues to solve difficult situations with patients. A participant had worked in the emergency department for 2 years and mentioned such experience:

*Sometimes women nurses are too soft with a low voice, and male patients do not listen to them, for example, refusing to move out of the emergency rooms. Our female colleagues come to us for help. We apply some manly approach, making demands. We are firm in our stance, “You have to move!”. Very often, our styles work.* (MN 4)

#### Same-sex health care

Male nurses were welcomed by some male patients, particularly when the health care was to be performed in the privacy areas of the body, such as the perineal areas. A new nurse who had worked for just 1 year told of a story: “*One of my relatives was an elderly man. He once was given urine catheterization by a female nurse. He was so embarrassing that he covered his eyes with a pillow the whole process*.” (MN 3). Male nurses sometimes acted as chaperon. “*If the physician is going to assess a patient of different sex. They need a third person. We call the third person ‘the flower vase’, just showing up there. We are sometimes ‘the flower vase’*.” (Laughing) (MN 15).

#### The pursuit of professional development as expected

Among the 12 male clinical nurse participants, four had completed postgraduate programs, such as master or postgraduate diploma program; six were undergoing or applying for postgraduate programs. Those who had had a postgraduate degree were contemplating or were undergoing further study, such as Ph.D. programs. The only two who had not considered further education were the new nurses with only 1 year of work experience.

Male nurses expressed that they bore higher expectations from the head-nurses as well as the society than female nurses. One male nurse in hemodialysis unit was applying for a postgraduate program in cardiac nursing, renal care, or psychiatric nursing. He talked about the male nurses’ pursuit of professional development as propelled by a mixture of inner motivation and the outside pressure:

*Males have advantages in professional development because female nurses pay more attention to their family than to professional development. Men are more ambitious in career development than women. I see that the man nurses in our hospital all go to advanced nursing programs. Even if they themselves do not apply, their superiors would suggest they apply. Traditionally people think that men should go high in their careers; the higher, the better.* (MN 14)

### Theme 3: taking an egalitarian viewpoint

The male participants emphasized that nurses were professionals and that qualified service was expected by patients and the whole society, regardless of the sex of the nurses. There are two subthemes relating to theme 3: “personality versus gender” and “increased acceptance”.

#### Personality versus gender

The junior nursing students agreed to a certain extent that females were gentler, more careful, more empathetic than males, and these traits could help with communications between nurses and patients. Women’s carefulness could prevent mistakes from happening, assuring safe health care. The senior students and the clinical nurses, based on their experiences, challenged the notion that women were superior to men to do nursing. A senior student who had finished Year 4 expressed his opinion that gender itself had no relation to quality of health care.

*Good health care does not depend on whether the nurse is careful or careless but depends on whether the nurse has put her/his heart into work. Mothers often say to their daughters: as a girl, you should be careful when doing things. I think carefulness doesn't exist by nature. It is developed through education and training.* (MN 3)

#### Increased acceptance

Male nurses’ sense of gender egalitarian was also shaped by increasing acceptance of the broad society to male nurses. The female-dominated workforce still marks Macau in nursing. This is reflected by the feminine title labeled to male nurses. Nurses were called “Gu-niang”, meaning “girl” in Macau and Hong Kong. The male participants all had been called “Gu-niang” by patients. Other titles were also applied to the male nurses, such as “girl-boy”, “nurse”, “sir”, etc. People had no idea what they should call male nurses. A male who grew up in Macau talked about the history of Gu-niang in Macau.

*Gu-niang means young women. Nurses were young women, so Gu-niang became the name of nurses exclusively. This has lasted for decades. I am called girl by patients, but that doesn’t matter. I don’t mind what the patients call me as long as they show respect to me. I know in Hong Kong people call male professionals “Sir”. They call male nurses and policemen “Sir”.* (MN 3)

Those male participants who had worked for a longer time than other participants had observed that more and more patients called male nurses “nurse” or “sir”. This, according to the experienced participants, reflected acknowledgment of nursing as a profession, as a senior nurse who had worked for 9 years explained why more people accepted male nurses:

*People may say men should do a decent job and support their families. Nowadays, nurses' social status has improved. Nurses have a bachelor’s degree. They are professionals. Thanks to mass media propaganda in Macau and Hong Kong, particularly during the SARS (Severe Acute Respiratory Syndrome) period in 2003, the public now know better about nursing than before. Nurses are not only doers, following the doctors' orders, but they also have their own specific knowledge and skills. Nursing is a decent job with reasonably paid and stable salaries that are well higher than middle levels in Macau. People are more open to male nurses.* (MN 20)

This man pointed several contributors to the enhanced social status of nurses in Macau. A positive social environment to male nurses had formed in Macau to increase men’s sustaining in nursing.

## Discussion

Male nurses in Macau are still a minority among nursing professionals, and they are still attached to a feminine title of “Gu-niang”. However, with the increasing acceptance of the public to nursing as a profession, a positive social image of nurses is forming, leading to a positive attitude towards male nurses among the public as well as a positive self-image among the local male nurses. While nurses build their professional identities in nursing education and nursing practice, they encounter both barriers and advantages in the process [26–28]. This study found that, by taking perceived advantages of masculinity, male nurses circumvented the barriers and took on their strengths to advance their professional development.

Previous studies found male students’ negative experiences in nursing, for instance, being marginalized by female counterparts [3, 12, 16]. Our study identified some similar negative experiences among male students. A subtle difference was observed that junior and senior students and clinical nurses held a different opinion on the gender-related nurse identities. Junior students somehow supported the lay knowledge of femininity being superior to masculinity in nursing. Other scholars revealed a low level of self-image among male nursing students [12, 16, 29]. Our study found that males became more positive to nursing as they knew more of nursing. Due to the limited number of participants in the study, such differences need to be verified in other qualitative and quantitative research studies.

Numerous studies have found embarrassment among patients when they are provided healthcare around privacy areas by the nurses of the opposite sex [3, 29–31]. The male nurses in this study showed their understanding of the objections from patients. On the other hand, they thought of ways to win over patients for cooperation. Male nurses’ ability to deal with their relationships with patients in different situations reflected their flexible coalition strategies.

Due to a lack of nursing workforce, male nurses were assigned to a limited number of clinical wards. However, this employment limitation tended to be an advantage for some males, as these wards provided more opportunities for masculine trait utilization [32, 33]. Particularly, the male nurses in our study challenged the traditional notion that the so-called feminine traits, such as gentle, empathetic, caring, were ideal for the nursing profession and that females possessed the feminine traits by nature. This implies men’s refusal to acknowledge that men are naturally inferior to women in the nursing profession.

Several studies reported that male nurses experienced isolation because they were a minority in the female-dominated profession [16, 29, 30]. Our study revealed the opposite phenomenon that male nurses enjoyed harmonious relationships with their female colleagues. Masculine traits in physiology and manly personalities enhanced men’ acceptance into the female-dominated nurse teams. This is supported by a study with Japanese male nurses in which the males gained support and recognition from their female counterparts by offering helps in physically demanding tasks, like lifting heavyweight patients [34]. The widely cited work by Williams [32] reported men’s gender-based rapport with female nurses because women actually welcomed men into “their” professions. Also, men can apply coalition strategies in establishment of relationships [3]. This is reflected in our study that the men were sometimes in a better position to deal with tough conditions in clinical contexts when nurse/patient conflicts arise.

### Implications for policy

Our study indicates that nursing students would experience barriers in their academic study. Nursing educators should offer more support for their students, who should have a say on teaching methods so that the teaching arrangements are more likely to be accepted by both male and female students. Studies have found that mass media have contributed to the female dominance of nursing because of a lack of male nurse images in their propaganda [3, 30]. Nursing schools can invite male students or male nurses in their recruitment advertisements. Nurses’ social image has an impact on the selection of nursing as the major among high school graduates. Again, nursing schools need to disseminate information to the high school students on the qualifications the nurses have and the roles they play in health systems [35].

Our study uncovered men’s endeavors in career development, as career success accommodates with masculinity [3, 11]. Furthermore, men are traditionally expected to be the breadwinners of their family, and low payment is the essential trigger for males to leave nursing [35]. Nurse-background legislators in Macau have always called for better social benefits for nurses. Nowadays, payment for nurses in Macau is well above the average income levels [17]. Nursing leaders in other places should act the same. The positive social image of nurse is another contributor to the commitment of male nurses to nursing. Other places should follow Macau’s example by carrying out propaganda in mass media and internets, as suggested by some scholars [35]. These propagandas have made male and female nurses in Macau visible to the public in a positive way.

### Limitations of the study

This study has inherent limitation with the qualitative research design because small sample size in qualitative research restricts the generalization of the research findings to other settings [36]. The study has two additional limitations. Firstly, despite the efforts from the researchers to recruit a heterogeneous sample, the participants came from one nursing school, restricting the generalization of the findings even further. Secondly, there are limitations with member check [37]. The researchers invited two participants to review the findings of the study. While this technique could enhance authenticity of the research findings, it has shortcomings. The researchers might have missed out different opinions from other participants than the two invited. No revision suggestions from the two participants might reflect they had provided information they believed the researchers wanted to hear.

## Conclusions

This study revealed males’ views about the advantages and disadvantages of being a male in the female-dominated nursing profession. Holding the egalitarian viewpoint, male nurses have taken on their masculinities to gain an equal position to female nurses. While our study echoed the common advantages and disadvantages with male nurses reported in other countries and regions, it revealed some findings supported by a limited but increasing number of studies, such as men’s coalition strategies to deal with the relationships with patients as well as female colleagues. The findings of the study support the calls from the International Nursing Association [38] for involvement of nurses in policymaking. The joining of male nurses may enhance the negotiating power for nursing interests in the current patriarchal health and governmental power system. An overall shortage of nursing workforce, together with the gender imbalance, is a long-term problem faced by all the countries and regions, and collective efforts from nurses in different places are needed. Our study’s findings have added evidence for developing strategies to tackle the common problem in the local region and beyond.

## Data Availability

The datasets used and/or analyzed during the current study are available from the first author and/or the corresponding author on reasonable request.

## Abbreviations

ICU: Intensive Care Units
WHO: World Health Organization
